# Dynamic Control of Neurotransmitter Release by Presynaptic Potential

**DOI:** 10.3389/fncel.2016.00278

**Published:** 2016-12-05

**Authors:** Mickael Zbili, Sylvain Rama, Dominique Debanne

**Affiliations:** UNIS, UMR_S 1072, Institut National de la Santé et de la Recherche Médicale (INSERM), Aix-Marseille UniversitéMarseille, France

**Keywords:** axon, sodium channels, synaptic transmission, brain circuit

## Abstract

Action potentials (APs) in the mammalian brain are thought to represent the smallest unit of information transmitted by neurons to their postsynaptic targets. According to this view, neuronal signaling is all-or-none or digital. Increasing evidence suggests, however, that subthreshold changes in presynaptic membrane potential before triggering the spike also determines spike-evoked release of neurotransmitter. We discuss here how analog changes in presynaptic voltage may regulate spike-evoked release of neurotransmitter through the modulation of biophysical state of voltage-gated potassium, calcium and sodium channels in the presynaptic compartment. The contribution of this regulation has been greatly underestimated and we discuss the impact for information processing in neuronal circuits.

## Introduction: Digital, Analog and Analog-Digital Signaling

**Figure 1:**
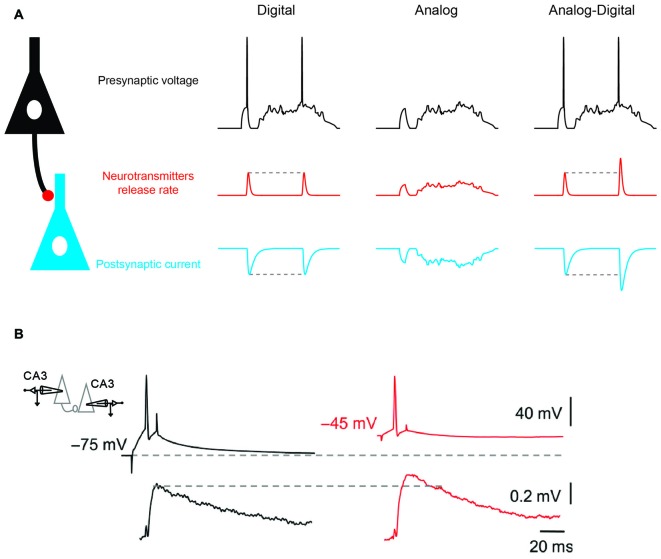
**Analog-digital (AD) mode of synaptic transmission. (A)** Digital, analog and hybrid (AD) modes of synaptic transmission. Left: digital mode of synaptic transmission. Transmission is stereotyped and occurs only if a presynaptic action potential (AP) is elicited. Middle: analog mode of synaptic transmission (graded transmission of presynaptic voltage fluctuations). Right: hybrid AD transmission. Both subthreshold fluctuations and spiking activity are transmitted. Note that when the presynaptic spike is produced after a prolonged period of depolarization (second spike) the spike-evoked synaptic response is enhanced compared with when there is no prolonged depolarization (first spike). **(B)** Depolarization-induced AD facilitation (d-ADF) of synaptic transmission at a CA3 –CA3 connection. Average excitatory post-synaptic potential (EPSP) traces (bottom) evoked by action potentials APs triggered from control (black traces) and depolarized presynaptic membrane potentials (red traces). Adapted from Bialowas et al. (2015).

Neuronal information in the mammalian brain is usually conveyed by action potentials (APs). The axon initial segment (AIS) expresses a high density of sodium channels, and therefore it constitutes a hot spot for generation of APs. Once initiated the spike propagates along the axon to the presynaptic terminals where it causes release of neurotransmitter. Neuronal information is thus transmitted to the post-synaptic neurons as discrete spike-evoked packets of neurotransmitter in an all-or-none mode of signaling. Thus, neuronal signaling is considered to be digital: if the spike threshold is crossed the neuron fires and generates an output but if the spike threshold is not reached no output is observed, and neurotransmitter release follows a binary mode of signaling (Figure 1A, left). Digital signaling presents several advantages. First, information is carried over long distances without dissipation because the AP is regenerated all along the axon (Debanne et al., 2011). Another advantage of digital signaling resides in its low energy cost. In fact, kinetics of voltage-gated sodium and potassium currents underlying the action potential are tuned to constrain energy consumption. The Na^+^ excess during APs is found to be close to the theoretical minimum (i.e., it varies between 1.3 and 2 fold, depending on axon type (Alle et al., 2009; Hallermann et al., 2012)). If digital signaling presents advantages, it has also limitations. The coding of information by a digital synapse is generally poor because of the discrete nature of digital signaling (Borst and Theunissen, 1999).

Neuronal information is not only transmitted in digital mode and subthreshold activity originating from the dendrites and the soma can be conveyed by the axon to the presynaptic terminal where the flow of information is coded in an analog mode (Figure 1A, middle). Pure analog transmission has been reported in invertebrate neurons and in the inner ear or in the retina of mammalians where sensory stimulation produces graded changes in membrane potential without APs (Werblin and Dowling, 1969; Heidelberger, 2007). These cells release transmitter in a tonic mode and their rate of release is a function of the presynaptic membrane potential. Compared to digital synapses, analog synapses display a much higher rate of information transfer (Borst and Theunissen, 1999). However, the energy consumption and voltage dissipation along neuronal processes represent two major drawbacks of analog signaling (Debanne et al., 2013).

It has been recently shown that analog signals modulate the function of digital synapses. In fact, subthreshold activity in the presynaptic element modulates spike-evoked transmission, leading to the emergence of the concept of hybrid analog-digital (AD) synaptic transmission (Figure 1A, right). Initially described in invertebrates (Takeuchi and Takeuchi, 1962; Kusano et al., 1967; Shimahara and Tauc, 1975), AD facilitation (ADF) of synaptic transmission has been reported in many mammalian synapses including cortical (Shu et al., 2006; Kole et al., 2007; Zhu et al., 2011; Rama et al., 2015a), cerebellar (Bouhours et al., 2011; Christie et al., 2011) and hippocampal synapses (Saviane et al., 2003; Alle and Geiger, 2006; Sasaki et al., 2012; Kim, 2014; Bialowas et al., 2015; Rama et al., 2015a). Most of ADF reported so far has been induced by long (0.3–10 s) subthreshold depolarization of the soma (Saviane et al., 2003; Alle and Geiger, 2006; Shu et al., 2006; Kole et al., 2007; Bouhours et al., 2011; Christie et al., 2011; Sasaki et al., 2012; Bialowas et al., 2015) and correspond to depolarization-induced ADF (d-ADF; Figure 1B). In the other few cases, ADF has been induced by a transient hyperpolarization (15–200 ms) before the action potential (Cowan and Stricker, 2004; Thio and Yamada, 2004; Rama et al., 2015a). This form of plasticity corresponds to hyperpolarization-induced ADF (h-ADF).

In both cases, the principle underlying ADF is that membrane potential fluctuations in the cell body is electrically transmitted by the axon over hundreds of micrometers to the terminals where they modulate the biophysical state of voltage-gated potassium, calcium or sodium channels (Alle and Geiger, 2006; Shu et al., 2006; Christie et al., 2011; Sasaki et al., 2012; Debanne et al., 2013; Rama et al., 2015a,b). Thus, these forms of ADF can be found only in local circuits such as L5-L5 synapses in the cortex or CA3-CA3 synapses in the hippocampus where both the short axonal distance and the limited number of branch-points represent favorable conditions to an optimal transmission of voltage to the presynaptic terminal (Sasaki et al., 2012). Long distance connections with many branch points such as CA3-CA1 synapses usually do not express ADF (Sasaki et al., 2012).

## Depolarization-Induced AD Facilitation (d-ADF)

Two mechanisms have been identified to account for d-ADF. The first mechanism relies on inactivation of shaker-type voltage-gated potassium channels (Kv1). Kv1 channels are present in the axon of L5 and CA3 neurons where they control the spike duration and subsequently, neurotransmitter release (Kole et al., 2007; Shu et al., 2007; Boudkkazi et al., 2011; Foust et al., 2011; Kim, 2014; Bialowas et al., 2015). Inactivation of Kv1 channel is, however, a very slow process and depolarizations of ~5–10 s are usually required to fully inactivate Kv1 channels and produce a significant (i.e., ~30%) increase in neurotransmitter release (Kole et al., 2007; Bialowas et al., 2015). Thus, slow oscillations of network activity such as up and down states usually occurring during slow-wave sleep may represent a physiological condition in which d-ADF occurs (Shu et al., 2006). However, up-states affect equally all neurons thus creating conjoint shifts in membrane potential of 10–20 mV in presynaptic and postsynaptic neuron. Therefore, during up-states the driving force of the excitatory post-synaptic potential (EPSP) is reduced by ~30%. One may thus propose that d-ADF rather constitutes a homeostatic process to compensate for the loss of driving force of the EPSP due to the up-state.

The second mechanism is based on the activation of voltage gated calcium channels (Cav) by the subthreshold depolarization. In the axon of cerebellar interneurons, slow subthreshold depolarizations have been found to activate P/Q type (Cav2.1) Cav channels thus producing an elevation in basal Ca^2+^ concentration and subsequently an increase in spike-evoked transmission (Bouhours et al., 2011). Because, these Cav channels are activated by high levels of depolarization, their contribution to d-ADF is limited to short axons such as cerebellar axons (Bouhours et al., 2011) or to very proximal synapses (Bialowas et al., 2015).

## Hyperpolarization-Induced AD Facilitation (h-ADF)

Analog modulation by changes in presynaptic membrane potential is not restricted to voltage-gated K^+^ and Ca^2+^ channels. In fact, in excitatory neurons, a large portion of the voltage-gated Na^+^ (Nav) current in the axon and presynaptic terminal is inactivated at rest. In the axon terminal or the axon proper from dentate granule cells or from L5 pyramidal neurons, the inactivated fraction of Nav channels may reach 70%–80% (Engel and Jonas, 2005; Hu et al., 2009; Schmidt-Hieber and Bischofberger, 2010). In comparison, somatic Nav channels display much less inactivation (~20% in cortical layer five pyramidal cells (Hu et al., 2009)). The origin of this difference in Nav channel inactivation is not well established but it may result from the nature of the subunits. Nav1.6 is principally found in the distal axon whereas Nav1.2 is found in the proximal part of the axon and in the soma (Hu et al., 2009).

**Figure 2:**
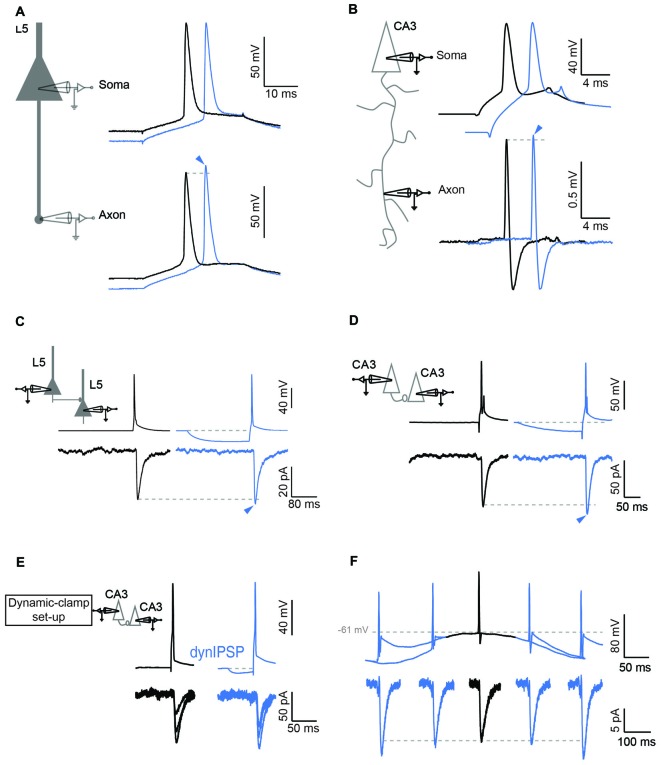
**Hyperpolarization induced ADF (h-ADF). (A,B)** Dual recording from the soma and the axon in L5 **(A)** and CA3 **(B)** pyramidal neurons. Left: scheme of experimental setup showing double recording from the soma and the axon. Right: soma–axon recording (in whole-cell in **A** and in cell-attached recording in **B**). Hyperpolarization of the soma (blue traces) enhances the spike amplitude measured in the axon but not in the soma. **(C,D)** Paired recording of synaptically connected L5 **(C)** and CA3 **(D)** pyramidal neurons. Note that a brief hyperpolarization of the presynaptic cell (blue traces) enhances the amplitude of the postsynaptic response. **(E)** Induction of h-ADF with presynaptic inhibitory post-synaptic potentials (IPSPs). Left: scheme of the dynamic-clamp system used to inject a current that mimics a GABAergic input in the presynaptic cell. Right: recording from a pair of CA3 neurons in the absence (black traces) and in the presence of a simulated GABAergic input injected into the presynaptic neuron before the spike (blue traces). The two groups of EPSCs are representative of the two conditions. Note that an IPSP waveform injection before the presynaptic spike enhances the amplitude of the postsynaptic response (blue traces). **(F)** θ oscillation induces h-ADF in CA3 neurons. Presynaptic APs are triggered at different phases of a subthreshold oscillation of the membrane potential at 4 Hz. h-ADF is observed when the spike is triggered in the trough of the oscillation. Adapted from Rama et al. (2015a).

The consequence of this elevated Nav channel inactivation in the axon is multiple. First, it may extend the spike initiation site to a wider axonal zone (Scott et al., 2014). But most importantly, it will largely modulate the amplitude of the action potential in the axon upon changes in membrane potential in the cell body (Rama et al., 2015a). In fact, it was shown in this study that hyperpolarizing the somatic potential enhanced the amplitude of the action potential recorded in the axon (Figures 2A,B). As a consequence, the spike-evoked calcium influx was found to be increased, and synaptic transmission was augmented (Rama et al., 2015a). This h-ADF was found to be present at both CA3-CA3 and L5-L5 connections (Figures 2C,D), suggesting that h-ADF might be a general feature in local brain circuits. Nav channel inactivation is a key factor in the expression of h-ADF because the increase in spike amplitude result from the recovery of Nav channel from inactivation. Thus, increasing Nav channel inactivation with carbamazepine or reducing the number of activatable Nav channels with tetrodotoxin (TTX) that subsequently enhances the modulation of the presynaptic spike amplitude by the hyperpolarization was found to augment the amplitude of h-ADF (Rama et al., 2015a).

h-ADF and d-ADF were found at the same connections and were found to be additive (Rama et al., 2015a). Compared to d-ADF, h-ADF is three orders of magnitude faster. Indeed, h-ADF can be induced by 15 ms hyperpolarization. This feature has important consequences in terms of network dynamics. First, h-ADF can be triggered by a hyperpolarizing inhibitory post-synaptic potential (IPSP; Figure 2E). In addition, h-ADF can be induced by 4 Hz theta oscillations (Rama et al., 2015a). The maximal facilitation was found to occur in the troughs of the oscillation (Figure 2F). Thus, in contrast with d-ADF, h-ADF adds on the post-synaptic modulation of the EPSP due to the increase in driving force during hyperpolarization of the network. *in vivo*, APs triggered in the troughs of theta oscillations are thought to improve the accuracy of spatial coding (O’Keefe and Recce, 1993). We suggest that phase-unlocked spikes would produce a stronger post-synaptic response through h-ADF, and hence would promote further the spatial coding. Finally, in a model of interconnected pyramidal cells and interneurons that expresses spontaneous gamma oscillations, addition of h-ADF was found to promote network synchrony at gamma frequency (Rama et al., 2015a).

## Conclusion and Future Directions

The recent identification of h-ADF adds a new form of plasticity in local circuits such as CA3-CA3 or L5-L5 synapses. As a fast process, h-ADF may impact the network properties during rapid activity regimes such as theta or gamma activity. In contrast, the impact of d-ADF on network properties is probably less important because of its slow kinetics. Rather, it can be seen as a homeostatic process that maintain stable synaptic strength during slow depolarization shifts. It will be important to incorporate these forms of short-term plasticity in realistic models of cortical circuits (Markram et al., 2015).

Will other forms of ADF be identified in the near future? There are serious reasons to believe that this will be the case. The study of functional properties of ion channels in the axon is only at its beginning. And the recent development of direct recordings from thin axons and presynaptic terminals (Novak et al., 2013; Kawaguchi and Sakaba, 2015; Begum et al., 2016; Rowan et al., 2016) together with the development of genetically-encoded voltage indicators (Hoppa et al., 2014) will certainly open new investigation opportunities about the role and function of ion channels in the presynaptic compartment.

## Author Contributions

MZ, SR and DD wrote the manuscript. MZ built the figures.

## Funding

Supported by Institut National de la Santé et de la Recherche Médicale (INSERM), Centre National de la Recherche Scientifique (CNRS), Fondation pour la Recherche Médicale (FRM) (doctoral grant FDT20150532147 to MZ) and Agence Nationale de la Recherche (ANR) (AXODE-14-CE13-0003-02).

## Conflict of Interest Statement

The authors declare that the research was conducted in the absence of any commercial or financial relationships that could be construed as a potential conflict of interest. The reviewer AD and handling Editor declared their shared affiliation, and the handling Editor states that the process nevertheless met the standards of a fair and objective review.
